# Lactate’s impact on immune cells in sepsis: unraveling the complex interplay

**DOI:** 10.3389/fimmu.2024.1483400

**Published:** 2024-09-20

**Authors:** Tao Zhang, Linjian Chen, Gatkek Kueth, Emily Shao, Xiaohui Wang, Tuanzhu Ha, David L. Williams, Chuanfu Li, Min Fan, Kun Yang

**Affiliations:** ^1^ Department of Biomedical Sciences, James H Quillen College of Medicine, East Tennessee State University, Johnson City, TN, United States; ^2^ Department of Surgery, James H. Quillen College of Medicine, East Tennessee State University, Johnson City, TN, United States; ^3^ James H. Quillen College of Medicine, East Tennessee State University, Johnson City, TN, United States; ^4^ Program in Neuroscience, College of Arts and Science, Vanderbilt University, Nashville, TN, United States; ^5^ Center of Excellence in Inflammation, Infectious Disease and Immunity, James H. Quillen College of Medicine, East Tennessee State University, Johnson City, TN, United States

**Keywords:** sepsis, lactate, lactic acid, lactylation, immune cells, inflammation, immune response, immunosuppression

## Abstract

Lactate significantly impacts immune cell function in sepsis and septic shock, transcending its traditional view as just a metabolic byproduct. This review summarizes the role of lactate as a biomarker and its influence on immune cell dynamics, emphasizing its critical role in modulating immune responses during sepsis. Mechanistically, key lactate transporters like MCT1, MCT4, and the receptor GPR81 are crucial in mediating these effects. HIF-1α also plays a significant role in lactate-driven immune modulation. Additionally, lactate affects immune cell function through post-translational modifications such as lactylation, acetylation, and phosphorylation, which alter enzyme activities and protein functions. These interactions between lactate and immune cells are central to understanding sepsis-associated immune dysregulation, offering insights that can guide future research and improve therapeutic strategies to enhance patient outcomes.

## 1 Introduction

Elevation in lactate levels is observed in a variety of critical illnesses, making lactate a useful biomarker for illness severity and prognosis (1, 2). In 1964, the use of lactate as a prognostic tool was first proposed by Weil et al. based on their pioneering observation that high lactate levels (> 4 mmol/L) correlated significantly with adverse outcomes in patients with shock (3). Since then, substantial clinical studies have been performed to establish the association between lactate levels and severity of sepsis (4, 5). Notably, the Third International Consensus Definitions for Sepsis and Septic Shock (Sepsis-3) has defined serum lactate level exceeding 2 mmol/L is as a clinical criterion in identifying patients with sepsis and septic shock (6). This consensus document has been considered as a milestone for inclusion of lactate in clinical guidelines and highlights lactate’s role in the pathogenesis of sepsis/septic shock.

Lactate has historically been known as a byproduct of glucose metabolism (7). Recent evidence has shown that lactate is an essential signaling molecule and epigenetic modulator, which plays a crucial role in the biological and pathological functions of different cells (8). The glycolytic pathway is central to this process, converting glucose to pyruvate within the cytosol of cells. Depending on the conditions, pyruvate either fuels the tricarboxylic acid (TCA) cycle for energy via oxidative phosphorylation (OXPHOS) or is converted to lactate by the enzyme lactate dehydrogenase A (LDHA) in the cytosol (9–11). With enough oxygen, pyruvate enters the TCA cycle, leading to the production of carbon dioxide and high-energy carriers for adenosine triphosphate (ATP) synthesis (12). In anaerobic conditions, cells convert pyruvate into lactate using LDHA to maintain ATP production albeit less efficiently (11, 13). Traditionally, we attribute the increased lactate levels to tissue hypoperfusion in patients with sepsis/septic shock (14). However, as our knowledge of pathogenesis of sepsis advances, it has become clear that other processes, not directly related to tissue oxygenation, may increase lactate production in sepsis/septic shock, such as activation of immune cells, impaired lactate clearance due to multiple organ injuries, and mitochondrial defects (15–18).

It is now recognized that sepsis is associated with a profound immunosuppression, which is a predisposing risk factor of nosocomial infection and mortality (19). Previous studies have demonstrated that lactate is a potent immunosuppressant in tumor microenvironments, thereby favoring tumor cell growth (20). Similarly, emerging evidence shows that lactate directly modulates the functions of a variety of immune cell, which contributes to immune paralysis in sepsis (21, 22). Despite the difficulty in targeting lactate production due to its complexity, targeting lactate receptor G protein-couple receptor 81 (GPR81) and the lactate transporter (MCT1) is suggested to restore immune responses in *in vitro* studies (21, 23).

In this present review, we focus on the molecular mechanisms by which lactate regulates immune responses during sepsis. First, we summarize the clinical recognition of lactate’s role in sepsis/septic shock patients. We then discuss the molecular and cellular mechanisms by which lactate determines the fate and behavior of immune cells in sepsis/septic shock. Last, we highlight the therapeutic potential of targeting lactate metabolism and lactate-associated signaling in treating sepsis/septic shock.

## 2 Recognition of lactate in sepsis/septic shock

### 2.1 Early observations

As early as 1843, Johann Joseph Scherer, a German physician-chemist, observed the presence of lactic acid in seven case reports of young women who died peripartum (24). These patients were diagnosed with perimetritis and with secondary peritonitis, hemorrhagic shock, or cerebral hemorrhage (24). Scherer hypothesized that the production of lactic acid was enhanced in such severe diseases. Scherer’s pioneering case reports are considered to be the foundational documentation of lactic acid as an indicator of septic and hemorrhagic shock, thereby paving the way for future exploration of lactic acid’s diagnostic and prognostic potential in various conditions.

During the 1960s -1980s, high lactate levels in patients with circulatory failure and shock were routinely observed in clinical practice (3, 4, 25–27). It was found that blood lactate levels indicate the severity of shock and offer a crucial prognostic index, effectively predicting outcomes even before the onset of severe hypotension (25). Also, these studies suggested that lactic acid is a major contributor to the metabolic acidosis observed in early shock. Lactate from venous blood in the right atrium, superior vena cava, or pulmonary artery are nearly identical to arterial levels, as shown by high correlation in studies (28). Huckabee suggested that a more precise evaluation of oxygen debt is achieved by measuring “excess lactate,” which refers to an imbalanced rise in lactate levels relative to pyruvate (29, 30). However, Weil et al. concluded that lactate levels alone may be a simpler and more sensitive prognostic indicator of the severity of shock (4). The study on 56 shock patients revealed an 89% mortality rate with lactate levels of 4 mmol/L or higher (3). Moreover, during fluid resuscitation of 17 patients with noncardiogenic shock, it was observed that the lactate concentration of 9 patients decreased by more than 5% within the first 60 minutes (31), suggesting that serial lactate tests during shock proved more reliable for prognosis. Although these early studies were not precise in terms of severe sepsis/septic shock, they did hold true for many of the subsequent studies (32, 33).

### 2.2 Recognition in sepsis

In the late 1980s, Cohen and Woods suggested that elevated lactate levels could result from inadequate oxygen supply (type A hyperlactatemia) or from factors unrelated to tissue hypoxia (type B hyperlactatemia) (34). Although seemingly simple, this rigid classification can be challenging to apply in complex clinical scenarios, particularly in the hyperlactatemia associated with sepsis, where it is categorized by some as type A and by others as type B (35). Indeed, this classification belie the complexity and breadth of the detailed kinetics involved in the production and utilization of lactate by tissues (35). Peripheral shunting (36) and heightened adrenergic stimulation (37) can also cause hyperlactatemia, but their prevalence and clinical significance in sepsis patients remain unclear. Despite this, lactate is a valuable marker for assessing tissue hypoxia and disease severity, importantly, independent on blood pressure (38). Research has found that blood lactate levels are more effective than oxygen-related metrics in predicting septic shock outcomes (39), with sequential blood lactate measurements being able to anticipate subsequent multiple organ failure (17). At that time, the significance of blood lactate concentrations equal to or exceeding 4 mmol/L in the context of early goal-directed therapy was acknowledged by clinical researchers (40, 41).

### 2.3 Inclusion in clinical guidelines

Lactate has appeared as a marker of hypoperfusion in the definition of severe sepsis and septic shock proposed in 1992 (42). In the first edition of the Surviving Sepsis Campaign (SSC) guidelines, lactate emerged as a measure of severity and symptomatic assessment of therapeutic endpoints (43). Guidelines suggest measuring serum lactate within 6 hours for suspected severe sepsis or septic shock patients. Lactate levels over 4 mmol/L indicate the need for early resuscitation therapy. Moreover, improved morbidity and mortality in severe sepsis and septic shock are linked to lactate clearance, aligning with the focus of SSC on treating tissue hypoperfusion early in resuscitation. Studies have shown that lactate clearance <10% has good specificity and sensitivity as an assessment to predict morbidity and mortality during hospitalization (15). Additionally, an analysis of the relevant database concluded that the guideline’s emphasis on measuring lactate provides tangible clinical benefits to patients (44). Subsequent studies have validated the idea that lactate can guide sepsis treatment (5).

### 2.4 Risk stratification

Accumulating evidence suggests the feasibility of applying lactate levels to sepsis risk stratification. For example, a retrospective multi-center study suggested that clinicians can utilize blood lactate concentrations greater than 0.75 mmol/L as an indicator to identify patients at an elevated risk of mortality (45). Patients in the emergency department with suspected infection and moderate lactate levels face a moderate to high mortality risk, even in the absence of hypotension (46). This suggests that lactate levels have substantial prognostic value in critical illness, including sepsis. Of note, it is reported that modifying treatments based on surrogate physiological targets from invasive catheter measurements is not essential for reducing mortality (47). This aligns with findings that serial blood lactate monitoring is equally effective as catheter-based measurements (5, 48). Moreover, research indicates that lactate clearance is associated with reduced mortality in critically ill patients, offering optimal prognostic utility for clinical application (1).

### 2.5 Integration into diagnosis and treatment algorithms

The pivotal role of lactate is reinforced in the Sepsis-3 definition of septic shock, which is distinguished from sepsis by the need for vasopressors to sustain a mean arterial pressure of 65 mm Hg or higher and a serum lactate level exceeding 2 mmol/L without hypovolemia (6). Lactate levels are also recommended for screening undifferentiated adult patients suspected of having sepsis, even when it’s not yet confirmed (49). Moreover, Gattinoni et al. (50) noted that understanding why lactate levels rise can lead to better treatment strategies, especially in deciding how aggressively to administer fluids to individuals. It was suggested that even if lactate levels do not fully normalize, values close to normal can signify effective resuscitation (51).

### 2.6 Ongoing research

In addition to being recognized as an important biomarker, lactate is also involved in the host immune responses by serving as a vital energy source for immune cells and other tissues in shock (52, 53). Recently, we and others have reported that lactate is a potent signaling molecule in mediating immune cell dysfunction and cardiovascular injuries in sepsis (21, 23, 54, 55). In addition, it has been reported that lactate regulates histone acetylation through inhibiting histone deacetylases, leading to altered gene expression (56). This observation highlights the role of lactate as an epigenetic modulator. In agreement, a recent study by Zhang and colleagues discovered that lactate induces a novel post-translational modification, named lactylation in which a lactyl group is added to lysine residues in histones (57). Lactate-induced histone lactylation differs not only in mechanisms from lactate-induced histone acetylation but also in the specific genes affected (57, 58). It is noteworthy that we and others have reported that lactate can promote the lactylation of non-histone proteins, such as high mobility group box 1 (HMGB1) and Snail1, in sepsis and other disease states (21, 55). This review examines the most recent advances in the mechanisms by which lactate regulates immune cell responses in sepsis/septic shock.

## 3 Sources of lactate in sepsis/septic shock

**Figure 1:**
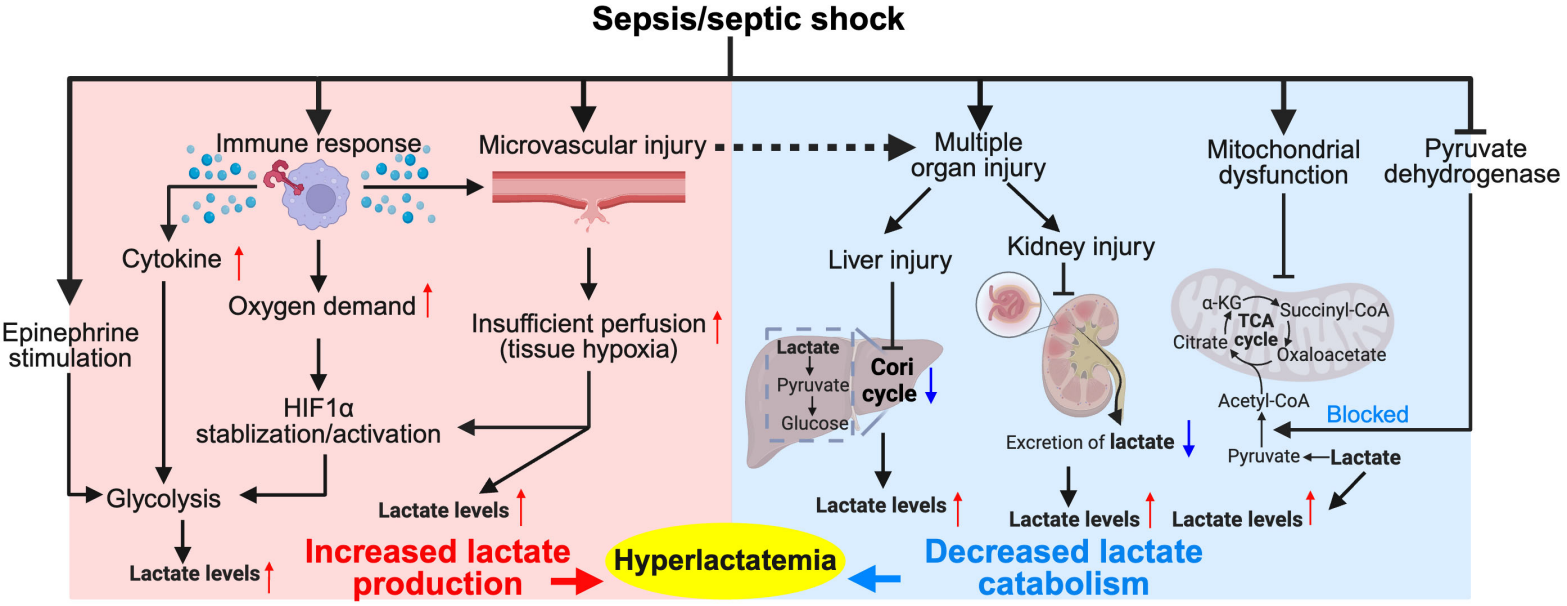
Major pathophysiological mechanisms of hyperlactatemia in sepsis/septic shock. The diagram illustrates the complex interplay between the immune response, tissue hypoxia, and metabolic alterations during sepsis and septic shock. Activation of immune cells leads to increased cytokine production, which along with epinephrine stimulation, enhances glycolysis and stabilizes hypoxia-inducible factor 1-alpha (HIF1α), resulting in increased lactate production. Tissue hypoxia due to microvascular injury further exacerbates lactate production. Concurrently, the Cori cycle in the liver and kidneys converts lactate back to glucose, but this process is often impaired in sepsis due to organ dysfunction, leading to decreased lactate catabolism and contributing to hyperlactatemia. This condition is further complicated by mitochondrial dysfunction, which impairs the conversion of pyruvate into the tricarboxylic acid (TCA) cycle intermediates, exacerbating lactate accumulation. Hyperlactatemia serves as both a marker of metabolic distress and a target for therapeutic intervention in sepsis management. Red colored arrows indicate activated events, while blue colored arrows indicate suppressed events, following sepsis/septic shock.

In sepsis and septic shock, lactate levels increase due to multiple factors (as shown in **Figure 1**). However, considering the potential overlap among individual causes, we lean towards a simplified classification based on the mechanism of lactate elevation: increased lactate production and impaired catabolism.

### 3.1 Increased lactate production

The human body constantly produces lactic acid (59), with levels spiking under increased cellular oxygen demand and/or reduced oxygen supply. In contrast to increased oxygen demand, hypoxia not only directly leads to lactate production, but also inhibits the degradation of Hypoxia-inducible Factor 1-alpha (HIF1α) and promotes its transcriptional activation (60). HIF-1α plays a multifaced role in regulating glycolysis and lactate production. It enhances glycolysis by inducing the transcription of glycolytic enzymes and membrane transporters, thereby increasing glucose flux (61, 62). Simultaneously, the expression of LDHA, a critical enzyme for lactate production, is heightened upon HIF-1α activation, leading to elevated lactate levels (63). During the hyperinflammatory phase of sepsis, increased oxygen consumption by activated immune cells leads to tissue hypoxia, which, in turn, stabilizes the transcription factor HIF-1α and consequently increases lactate production (64–66). For example, changes in glycolytic metabolism, induced by Toll-like receptors (TLRs), are key to activating dendritic cells, with anaerobic ATP production proving beneficial in low-oxygen conditions typical of infection/inflammation (67). This is consistent with a previous report that HIF-1α boosts lipopolysaccharide (LPS)-induced glycolysis in dendritic cells (68). Therefore, increased oxygen demands during inflammatory responses underscores a critical adaptation of activated immune cells to HIF-1α-dependent elevation of lactate production.

In the early phase, sepsis is characterized by a pronounced surge of the pro-inflammatory cytokines (69). Numerous studies have indicated that pro-inflammatory cytokines, especially interleukin (IL)-1β, are critical mediators in aerobic glycolysis and lactate production (70–73). Palsson-McDermott et al. demonstrated that TLR4-mediated tetramerization of pyruvate Kinase M2 (PKM2) promotes the transcription of IL-1β, leading to enhanced lactate production in LPS-activated macrophages (74). In addition, other pro-inflammatory cytokines, including IL-2, IL-3, IL-7, interferon-γ (IFN-γ) and tumor necrosis factor-α (TNF-α), also enforce the glucose metabolism and lactate production (71–73). These observations are in line with a previous study showing that lactate production in sepsis may be attributed more to inflammation rather than serving solely as a marker of tissue hypoxia (75).

It is noteworthy that enhanced lactate production in response to infection is a ubiquitous phenomenon and can occur in nearly all cells during sepsis. Early T cell activation (minutes to hours) increases aerobic glycolysis and diverts pyruvate to lactate production in a T cell receptor (TCR)-dependent mechanism (76). Neutrophils exhibit high glycolytic activity with limited mitochondrial respiration (77, 78). It is reported that human neutrophils can secrete lactic acid via a monocarboxylate transporter (79). In a murine model of acute inflammation, it is further illustrated that nicotinamide adenine dinucleotide phosphate (NADPH) oxidase (NOX)/reactive oxygen species (ROS)-mediated HIF-1α activation is required for lactate production in activated neutrophils (80). While activated immune cells are recognized as a primary source of lactate production during sepsis, the condition also induces upregulation of glycolysis and lactate production in numerous other cells and tissues. Endothelial cells (ECs) constitute the inner cellular lining of the blood vessels (81). Emerging evidence indicates that activated ECs rely heavily on glycolysis rather than on OXPHOS for ATP production during immune responses due to the low mitochondrial content, which consequently enhance lactate accumulation (82–85).

### 3.2 Impaired catabolism of lactate

If a large amount of lactic acid accumulates in the body, lactic acidosis will ensue (86). In response, efficient mechanisms are required for its clearance. The homeostasis of lactate is primarily maintained through its catabolism, which involves the conversion of lactate into pyruvate through the lactate dehydrogenase B (LDHB) enzyme (87). Pyruvate then enters the TCA cycle in mitochondria for further oxidation and energy production through pyruvate dehydrogenase (PDH), contributing to irreversible lactate removal (88). In severe sepsis, mitochondrial dysfunction (16, 89) and pyruvate dehydrogenase dysregulation (90–93) decrease OXPHOS, which interferes with the TCA cycle. This, in turn, accelerates lactate accumulation in sepsis. Intriguingly, emerging evidence from both clinical and pre-clinical studies indicates the activity of PDH is decreased in sepsis (90, 94, 95). It is reported that PDH activity and quantity are significantly lower in the peripheral blood mononuclear cells of patients with sepsis than the control group (90). Further analysis showed that the level of PDH activity is lower in sepsis non-survivors when compared to survivors (90). Importantly, an inverse association between baseline lactate levels and PDH activity in these patients, suggesting that PDH dysregulation contributes to enhanced lactate levels in sepsis (90). In a rat model of sepsis, induced by intraperitoneal inoculation of *Escherichia coli* and *Bacteroides fragilis*, active form of PDH is decreased by 70% in skeletal muscle (94). A recent study also reports that decreased PDH activity in endothelial cells leads to lactate production and endothelial injuries in LPS-induced sepsis (95). Mechanistically, sepsis stimulates the activation of pyruvate dehydrogenase kinase (PDHK), which negatively regulates PDH activation via inhibitory phosphorylation (96).

In addition, excess lactate can be resolved via the Cori cycle or lactic acid cycle (97). Circulating lactate shuttles to the liver, where it is reutilized by hepatocytes through gluconeogenesis to form glucose again (97). Also, it can be oxidized and removed by different tissues or secreted into the urine via the kidneys. However, we must acknowledge that the phenomenon of hepatic and renal dysfunction is not uncommon in severe sepsis and septic shock, which could potentially contribute to hyperlactatemia in sepsis. This notion is supported by the observation that higher lactate levels correlate with higher Sequential Organ Failure Assessment (SOFA) and quick SOFA (qSOFA) scores (98, 99). Other conditions, such as peripheral shunting (36) and heightened adrenergic stimulation (37) can also cause hyperlactatemia, but their prevalence and clinical significance in sepsis patients remain unclear.

## 4 Lactate regulates immune cell function in sepsis

**Figure 2:**
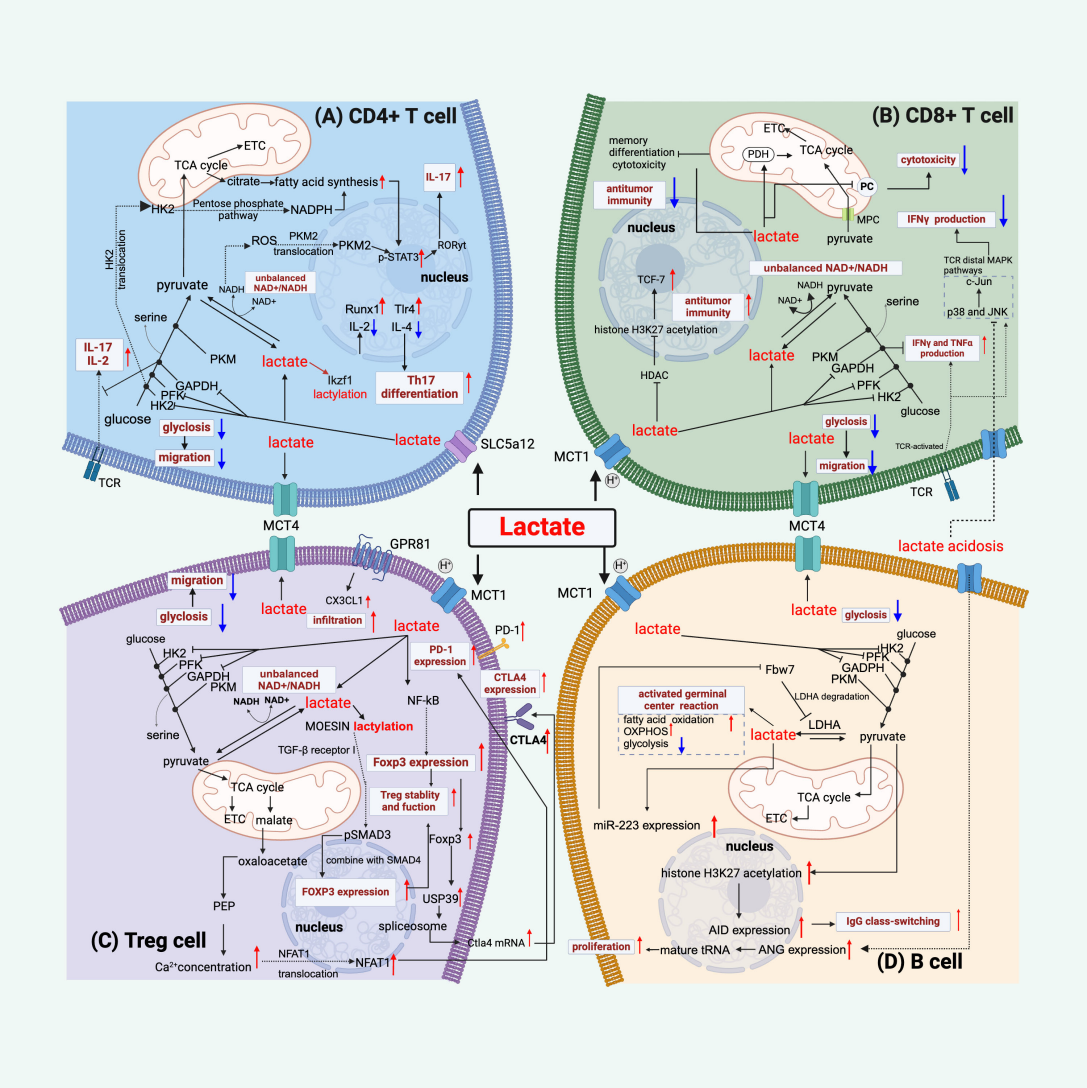
Lactate regulates lymphocyte function. **(A)** Lactate regulates CD4^+^ T cell function by the following mechanisms: inhibiting glycolysis and thus migration, disrupting the balance between NAD^+^ and NADH, mediating an increase in STAT3 phosphorylation by affecting PKM2 nuclear translocation and facilitating fatty acid synthesis, thereby increasing IL-17 production, and regulating gene expression by lactylation of Ikzf1, which facilitates differentiation to Th17 cells. **(B)** Lactate modulates CD8^+^ T cells by mechanisms such as, inhibiting glycolysis and thus migration, disrupting the balance between NAD^+^ and NADH, affecting pyruvate metabolism and thus reducing cytotoxicity, inhibiting histone deacetylase (HDAC) and thus promoting acetylation of histone H3K27 and thus increasing anti-tumor immunity, and its inhibition of the glycolytic enzyme GAPDH contributes to the production of IFN-γ, but appears to inhibit TCR-mediated IFN-γ production. **(C)** For Treg cells, in addition to limiting glycolysis, lactate binding to GPR81 enhances their infiltrative capacity, it improves PD1 expression by increasing the NAFT1 nuclear translocation mechanism, and it mediates high CTLA4 expression by up-regulating Foxp3, and lactate also promotes the lactylation of MOESIN to improve Treg function. **(D)** Lactate acts on B cells by the following mechanisms: promotion of germinal center function, inhibition of glycolysis, promotion of proliferation through increased ANG expression or positive feedback through miR-223-mediated lactate production, and lactate mediates the acetylation of histone H3K27 and thus promotes IgG class switching. Red colored arrows indicate activated events, while blue colored arrows indicate suppressed events, by lactate during sepsis/septic shock.

**Figure 3:**
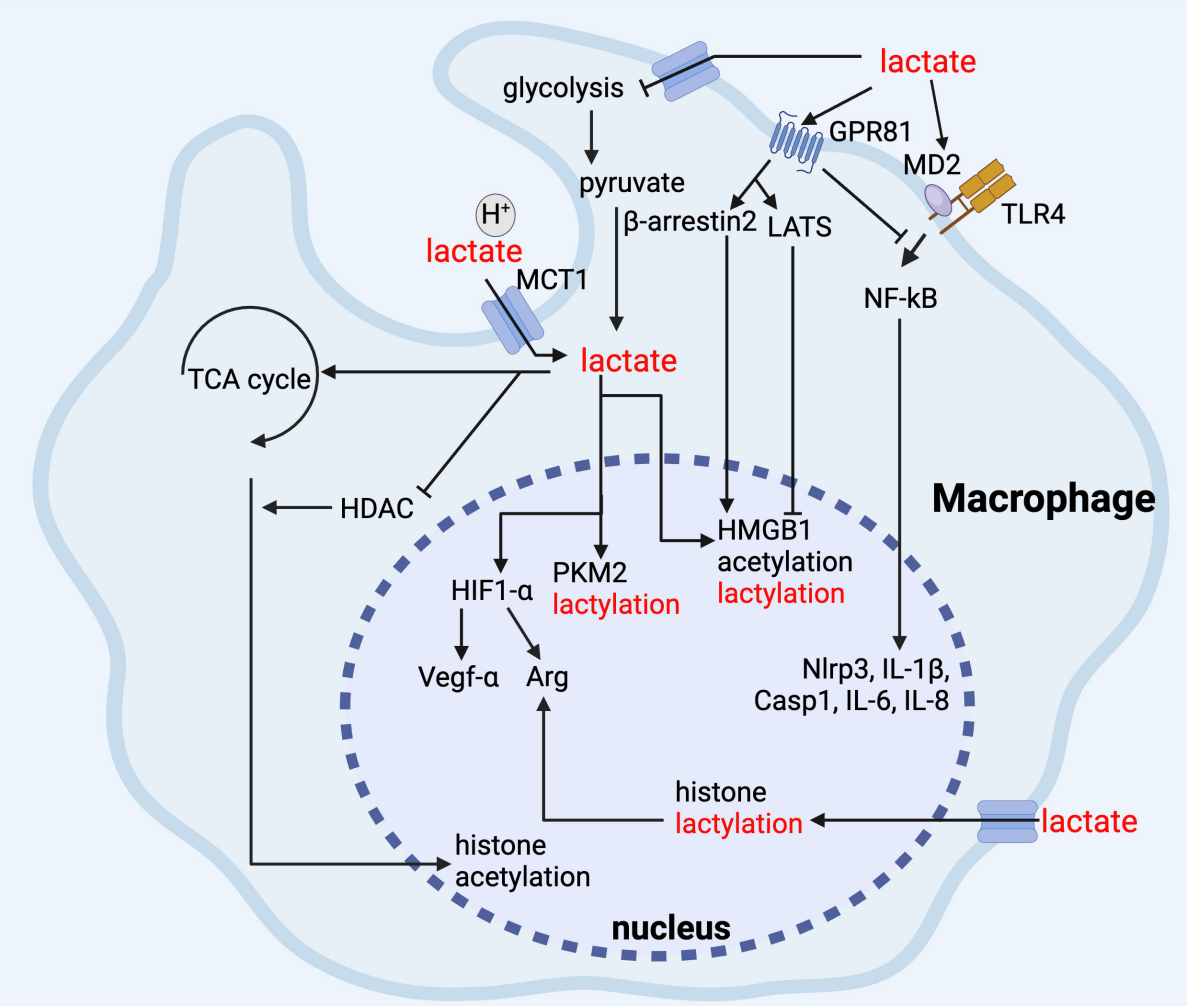
Lactate regulates macrophage function. In macrophages, lactate inhibits glycolysis, and its binding to GPR81 inhibits TLR-mediated pro-inflammatory responses via NF-kB, and it promotes Arg-1 and Vegf-α gene expression via up-regulation of HIF-1α, and the elevation of Arg-1 promotes histone lactylation, and lactate maintains its anti-inflammatory function via inhibition of HDAC and promotion of histone acetylation via TCA cycling. In addition, lactate can maintain anti-inflammatory function by inhibiting HDAC and promoting TCA cycle to promote histone acetylation, whereas for non-histone proteins, such as HMGB1, lactate can mediate lactylation and acetylation through β-arrestin2 promotion of p300/CBP and LATS/YAP-mediated inhibition of SIRT1.

**Figure 4:**
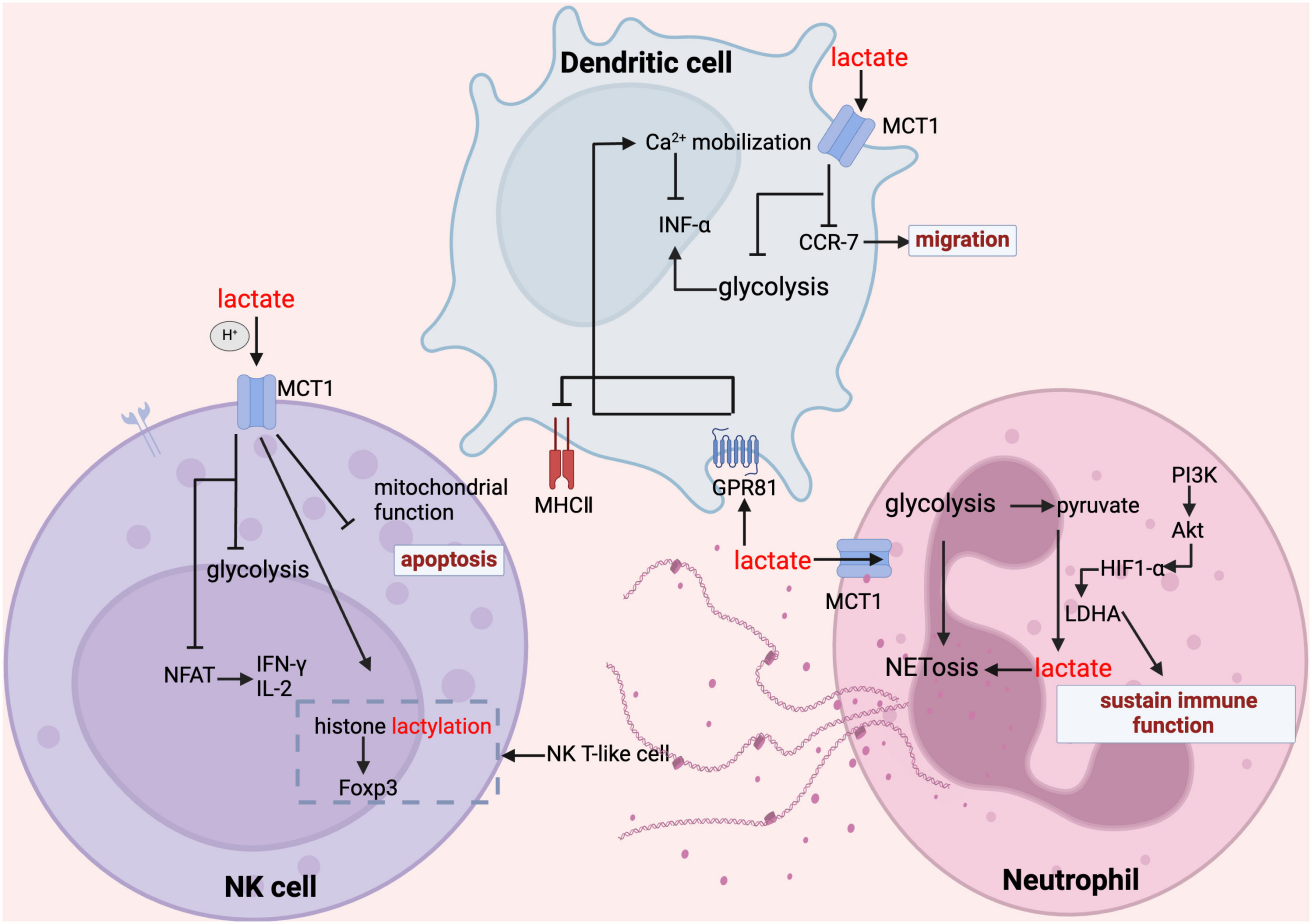
Lactate regulates NK cell, dendritic cell and neutrophil function. In NK cells, lactate inhibits glycolysis, suppresses mitochondrial function, inhibits the expression of NFAT and NKp46, promotes apoptosis and attenuates their ability to secrete inflammatory factors; whereas, for NK-T cells, the increase in histone lactylation promotes the expression of Foxp3. In dendritic cells, lactate inhibited glycolysis, inhibited CCR-7 and thus migration, lactate inhibited MHC II function through GPR81 signaling, and in addition GPR81-mediated Ca2+ mobilization inhibited IFN-α secretion. In mesangial cells, lactate maintains their immune function and promotes their NET formation.

In sepsis, lactate plays a pivotal role in regulating immune cell functions and metabolic processes (**Figures 2**–**4**). This regulation occurs through multiple mechanisms. Lactate interacts with immune cells via specific receptors and transport mechanisms. Primarily, lactate transport across cell membranes depends on monocarboxylate transporters (MCTs), particularly MCT1 and MCT4 (21, 100). MCT belongs to the solute carrier 16 (SLC16) family (101). MCT1, encoded by the SLC16A1 gene, primarily facilitates the uptake of lactate into cells, including liver cells for gluconeogenesis (102–104). MCT4, found in glycolytically active cells, predominantly manages the export of lactate, crucial for maintaining high rates of glycolysis, and its expression is upregulated via the myeloid differentiation primary response 88 (MYD88)/nuclear factor kappa-light-chain-enhancer of activated B cells (NF-kB) pathway (105, 106). Moreover, sodium-conjugated lactate can be transported by SLC5A8 and SLC5A12 (107). Indeed, the unique effects on CD8^+^ and CD4^+^ T cells are determined by the distinct expression of MCT1 and SCL5A12, respectively (108).

The main receptor for lactate is the GPR81, also known as hydroxycarboxylic acid receptor 1 (HCAR1), which plays a significant role in mediating lactate’s effects on energy and lipid metabolism, neuron protection, and inflammation (105, 109–118). In critical illnesses, such as sepsis and cancer, where immune dysregulation is prevalent, understanding the lactate mediated signaling is essential (20, 98, 119–121). Accumulating clinical and pre-clinical evidence has suggested that lactate is a potent modulator of immune responses by influencing both the activity and the metabolic regulation of immune cells (122). A prospective cohort study in septic shock patients found that changes in lymphocytic mitochondrial metabolism correlate with post-resuscitation arterial lactate levels but not with hypoperfusion status (123). Additionally, lactate regulates immune cell function through post-translational modifications such as lactylation, acetylation and phosphorylation, alter the activities of enzymes and the functions of various proteins (21, 23, 124). In sepsis-associated lung injury, histone lactylation-regulated methyltransferase-like 3 (METTL3) promotes ferroptosis (125). This complex interplay highlights the importance of lactate in immune regulation during sepsis, underscoring its potential as a target for therapeutic intervention.

### 4.1 Lymphocytes

The impact of sepsis and septic shock on T and B lymphocytes has been reported previously (126–129). Davie et al. (126) found that lower immunoglobulin G1 (IgG1), IgM, IgA in B cells and disrupted T cell ratios at Intensive Care Unit (ICU) admission signaled poor outcomes, while sepsis brings fluctuating immunoglobulin levels and worsening B/T cell changes. In addition, septic shock further depletes B cell IgM and alters T cell markers, with post-mortem findings of reduced lymphoid structure and T cell function, revealing profound immune suppression (126). However, understanding the origins and progression of lymphopenia remains a significant knowledge gap in the immunology of pre-sepsis and sepsis (130). It is intriguing that a growing corpus of evidence underscores the significance of lactate in this context.

T cells encompass various subsets, and distinct T cell subsets have different metabolic characteristics. Our discussion focuses on effector T cells, specifically CD4^+^ and CD8^+^ T cells, along with regulatory T (Treg) cells. In sepsis, all effector T cells except Treg cells decline (127), coupled with studies suggesting T lymphocyte exhaustion as a central aspect of the septic immunosuppressive effects (128, 129).

At the cellular level, lactate impacts the function and quantity of effector T cells. It inhibits glycolysis and restricts effector T cell proliferation through redox changes and reductive stress, independent of acidity (131–133). Recent research has shown that lactic acid undermines T cell function by weakening the T cell redox system through a reduction in oxidant and antioxidant molecule production (134). Additionally, lactic acid will accumulate and inhibit the differentiation of T cells *in vitro* (132). However, this effect appears to be pH-dependent, as normalizing the pH levels restores T cell function (131).

In sepsis, elevated expression of programmed cell death protein-1 (PD-1) or its ligand PD-L1 impairs T cell function, increasing mortality (135, 136). Targeting these molecules has shown promise in mitigating sepsis-induced immunosuppression by improving T cell efficacy (137, 138). Cellular signaling pathway studies revealed that lactate’s role extends to promoting PD-1 expression via nuclear factor of activated T cells1 (NFAT1) translocation by GPR81 signaling (139), then inducing effector T cell apoptosis and impairing cytotoxicity (140, 141). Elevated PD-1 and higher PD-L1 on antigen-presenting cells mark immune suppression beyond T cells, making lactate/lactate-associated signaling a valuable target. Recent research supports that lactate can induce a polarization of the effector phenotype of CD4^+^ T cells, which can lead to more IL-17 and IL-2 production (108, 142), and of CD8^+^ T cells, resulting in increased IFN-γ production (142). This increase is dependent on the activation of CD3/T cell receptor (TCR) signaling.

At the metabolic level, lactate can serve as an alternative carbon source for T cells, supporting its metabolism, and fostering polarization and activation (143, 144). Interestingly, lactate stimulates the mitochondrial electron transport chain without being metabolized, which boosts naive T cell proliferation and then their effector capabilities (145). In addition, lactate can induce metabolic reprogramming of T cells by enhancing fatty acid synthesis. This is mediated by nuclear PKM2/signal transducer and activator of transcription 3 (STAT3) signaling (146). High lactate can also mediate pyruvate shunt by affecting pyruvate carboxylase activity in CD8^+^ T cells, which in turn contributes to their decreased cytotoxicity (147). Moreover, lactate is reported to promote memory phenotype differentiation in CD8^+^ T cells by interfering with mitochondrial pyruvate metabolism (148) and supports TCA cycle anaplerosis in effector T cells (143). Interestingly, HIF-1α-induced mitochondrial metabolic reprogramming may be responsible for persistent infection-associated T cell exhaustion (149). However, the significance of lactate in relation to this mechanism requires thorough evaluation. Notably, immune cells undergo metabolic changes during both the hyperinflammatory and immunotolerant stages of sepsis, highlighting the significance of lactate in these processes.

At the epigenetic level, lactate-induced lactylation plays a crucial role in CD4^+^ T cell differentiation. Specifically, high lactylation on the transcription factor Ikzf1 enhances T helper type 17 (Th17) cell differentiation by directly influencing the expression of Th17-related genes, such as *Runx1, Tlr4, IL-2, and IL-4* (150). Lactate can suppress histone deacetylase activity which results in increased acetylation of Tcf7 (56) then can enhance the function of anti-tumor immune (151) and stemness of CD8^+^ T cells (152). Understanding these epigenetic mechanisms could provide insights into new therapeutic strategies for modulating immune responses in sepsis.

Regulatory T (Treg) cells play a vital role in the pathogenesis of critical illness, including sepsis and cancer, through their immunosuppressive functions (153, 154). Treg cells persistently increase during the late phase of sepsis (155), which contributes to the impaired immune responses in sepsis. A key aspect of their immunosuppressive role is that Treg cells can thrive in high lactate conditions without losing proliferative or functional capacity (156, 157). Mechanistic studies revealed that lactate mitigates the harmful effects of high glucose on Treg cells (158). In addition, lactate is reported to promote the transformation of naive T cells into Treg cells by activating NF-kB signaling and upregulates Foxp3, which supports the expansion of Treg cell populations and suppresses T cell pathogenicity (159, 160). In tumor microenvironment, lactate bolsters Treg cell stability and function by promoting MOESIN lactylation (161). A recent study has shown that lactate treatment enhances MOESIN lactylation, which boosts its interaction with TGF-β receptor I, and lactate also activates downstream TGF-β signaling through SMAD3 phosphorylation, leading to enhanced differentiation of Treg cells (161). I believe that this partly explains the mechanisms of combining anti-PD-1 with reduced lactate production by LDH inhibitor is more effective than anti-PD-1 alone (161). Since cancers and sepsis share similar immunosuppressive profiles, suppressed lactate production, which reduces Treg cell induction, is noteworthy. Cytotoxic T lymphocyte antigen 4 (CTLA-4) is a crucial immune checkpoint receptor that inhibits the activation and proliferation of T cell in sepsis (162, 163). Pre-clinical studies demonstrated that treatment with anti-CTLA-4 antibody maintains effector T cell function and improves sepsis survival outcomes (162, 164). Notably, lactate enhances the expression of CTLA-4 in tumor-infiltrating Treg cells via altering RNA splicing (165). It is intriguing to investigate whether lactate modulates CTLA-4 expression in Treg cells, potentially impairing immune responses in sepsis.

Sepsis is associated with significant B cells depletion due to apoptosis (166). An observational study revealed that the depletion of memory B cell populations played a role in sepsis-induced immunosuppression (167). B cells not only contribute to antibody production, but crucially enhance cytokine responses and bacterial clearance via communications with other immune cells, such as macrophages (168–171). Therefore, the decline in B cell numbers and impaired B cell function are considered as a prognostic biomarker for sepsis deterioration (171–174). Particularly, innate response activator (IRA) B cells are key in regulating inflammatory responses and managing sepsis outcomes, with their dysfunction linked to increased mortality, marking them and their produced IL-23 as potential targets for therapy (175, 176). Also, in sepsis, macrophage inflammation is intensified and lipolysis in adipose tissue is hindered due to the age-associated accumulation of B cells (170). However, compared to T cells, our understanding of the regulatory effects of lactate on B cells is relatively limited. B cells generate antibodies through germinal center (GC) and extrafollicular reactions (177). A recent study indicates that LDHA knockout in B cells hinders GC formation and antibody responses (178). And high lactate ensuring pyruvate for H3K27 acetylation, crucial for IgG class-switching (179).

Beyond direct evidence, the sophisticated mechanisms of lactate metabolism in lymphoma research may provide new insights for sepsis research. Increased levels of LDH are correlated with a higher mortality rate in B cell lymphoma patients (180, 181). Serum LDH is a key marker for aggressive non-Hodgkin lymphoma and is one of the factors listed in the International Prognostic index (182, 183). Moreover, lactic acid promotes B cell proliferation (184, 185), either by cleavage of mature tRNA at the anticodon loop via enhanced angiogenin (ANG) expression (186), or by inducing miR-223 expression to target Fbw7 (187). Additionally, blocking MCT exposes the therapeutic potential for virus-induced lymphomas (188).

### 4.2 Macrophages

Macrophages are widely distributed and present in almost all tissues and organs performing various functions, primarily maintaining internal environmental balance and resisting the invasion of pathogens (189). The unique characteristic of macrophages is their ability to polarize into different phenotypes, such as the M1-like macrophages and M2-like macrophages, in different microenvironments (190). M1 macrophages exhibit a pro-inflammatory phenotype, which can release various inflammatory cytokines and promote the inflammatory response. In contrast, M2 macrophages display an anti-inflammatory phenotype capable of producing an anti-inflammatory response and repairing damaged tissues (191). In sepsis-induced immunosuppression, macrophages exhibit altered cytokine secretion with decreased levels of TNF-α, IL-1β, and IL-12, and increased levels of TGF-β, IL-10, and macrophage migration inhibitory factor (MIF) (192). In addition, antigen presentation is reduced in the immunosuppressive stage of sepsis, as evidenced by lower human leukocyte antigen-DR isotype (HLA-DR) expression and decreased antigen uptake (192).

Substantial evidence has demonstrated that lactate exerts several inhibitory effects on pro-inflammatory (M1) macrophages, including hindering their migration, glycolysis, inflammasome assembly, and chemokine and cytokine secretion (122). Activation of TLRs stimulates the production of pro-inflammation cytokines and induces the polarization of macrophages towards M1 phenotype. Notably, we and others have shown that lactic acid reduces LPS-induced production of pro-inflammatory cytokines in macrophages (193) and promotes macrophage polarization into M2 macrophages (194). Indeed, it is found that lactic acid promotes the transcription of genes associated with M2 macrophage polarization, a process reliant on MCT function, HIF-1α activation, and induction of inducible cyclic adenosine monophosphate (cAMP) early repressor (ICER) (195–198). Our recent study also revealed that lactic acid suppresses NF-κB p65 nuclear translocation, a typical inflammatory signal (23, 194), via GPR81 signaling in macrophages (23, 80, 199). Additionally, lactate disrupts the assembly of TLR-4-mediated NLR family pyrin domain containing 3 (NLRP3) inflammasome and IL-1β secretion in a GPR81-dependent mechanism in macrophages (113). Given that lactate preferably promotes M2 macrophage polarization and inhibits M1 polarization, it is hypothesized that in sepsis, elevated lactate levels could worsen the immunosuppressive state by driving macrophages toward an anti-inflammatory M2 phenotype. This shift may further impair pathogen clearance and weaken pro-inflammatory responses, exacerbating sepsis-induced immunosuppression. Further investigation into targeting lactate metabolism in macrophages could offer therapeutic potential in sepsis.

Lactate can also regulate the function of macrophages by serving as a critical mediator in the metabolic-epigenetic link (57, 200, 201). Previous studies indicate that lactate is a primary carbon source for histone acetylation, significantly influencing epigenetic modifications (202–204). Shi and colleagues recently discovered that lactate fuels histone H3K27 acetylation, enabling the expression of immunosuppressive genes like *Nr4a1*, thus transcriptionally repressing macrophage pro-inflammatory functions (204). This histone acetylation leads to LPS tolerance and results in long-term immunosuppression. In addition, lactate-induced lactylation of histone H3 lysine 18 residue (H3K18la) is found to increase the production of inflammatory cytokines such as IL-2, IL-5, IL-6, IL-8, IL-10, IL-17, IFN-α, and arginine (Arg) in patients, thereby accelerating the development of an anti-inflammatory response of macrophages in sepsis (205). As lactyl and acetyl groups both stem from glucose metabolism and share regulatory enzymes, there might be a dynamic equilibrium between histone lactylation and acetylation (206). However, the exact nature of this equilibrium is not fully understood. Of note, H3K18la levels correlate with SOFA scores, ICU stay time, and lactate levels, suggesting that H3K18la is a potential biomarker for the diagnosis and prognosis of septic shock (205). However, additional clinically relevant data are still required to substantiate this. Histone lactylation also aligns with inflammatory markers in sepsis, as confirmed by other studies (205, 207).

Lactate-induced non-histone lactylation modifications also have inevitable impacts on macrophage functions in sepsis. High mobility group box 1 (HMGB1) is a non-histone DNA binding protein, which can be released into extracellular environment and as a late mediator of endotoxin lethality in sepsis (208–210). As a DAMP, extracellular HMGB1 is tightly associated with several types of cell death in sepsis, including apoptosis, autophagy, pyroptosis, and ferroptosis, which can deeply influence macrophage function (209, 211–217). Our recent study demonstrated a novel role of lactate in promoting HMGB1 lactylation and acetylation within macrophages, leading to consequent release of HMGB1 via exosome secretion in polymicrobial sepsis (21). It is also reported that the lactylation of PKM2 critically hampers glycolysis and shifts macrophages towards a repair-oriented phenotype (218). This change is marked by an increased expression of Arg-1, which supports wound healing, thus highlighting PKM2 as a pivotal regulator of macrophage metabolic adaptations (218). The role of lactate in promoting phosphorylation is also important. We have demonstrated that lactate reduces TNF-α and IL-6 levels in LPS-stimulated macrophages by inhibiting NF-κB and yes-associated protein (YAP) activation (23). Mechanistically, lactate triggers the activation of AMPK and LATS1 in a GPR81-dependent manner, leading to YAP phosphorylation and its subsequent degradation (23).

While the previous discussion highlights lactate’s inhibitory effects on macrophage activation, it is also important to recognize that lactate paradoxically exerts pro-inflammatory effects. It is reported that lactic acid triggers the production of IL-23 in peripheral blood mononuclear cells (PMBCs) in the presence of LPS stimulation, which may subsequently stimulate lymphocyte activation (219). Similarly, Samuvel et al. found that lactate activates myeloid differentiation factor 2 (MD2), a co-receptor for TLR4, which intensifies the TLR4-mediated pro-inflammatory response and increases NF-κB pathway-dependent gene transcription in human macrophages (220). Mechanistic study further revealed that the lactate-enhanced TLR4 signaling activation is mediated by lactate transporter MCT (220). Furthermore, studies emphasize that lactate can fuel a specialized glycolytic process in macrophages, reliant on the enzyme PFKFB2, which, when activated by efferocytosis, continuously supports further efferocytosis (221). Therefore, further refined experiments are necessary to clarify this discrepancy.

### 4.3 NK cells

Natural killer (NK) cells are effector lymphocytes of the innate immune system. They are uniquely primed for rapid and non-specific innate immune response against infections, without the need of antigen-presenting cells or prior exposure to pathogens (222, 223). As the primary innate lymphocyte population, NK cells are pivotal in orchestrating early responses to bacterial infections. Although they bolster the antimicrobial functions of myeloid cells, especially macrophages, by producing interferon-γ (IFN-γ) (222), a large number of animal experiments and human-related studies have confirmed the deleterious effects of overwhelming activation of NK cells in acute sepsis (224–227). It is noteworthy that sepsis rapidly induces phenotypic alterations and extensive cellular apoptosis in various types of immune cells, including NK cells, leading to profound immune paralysis (228–232). Single-cell RNA sequencing revealed downregulation of cytotoxic genes in NK cells among late-stage sepsis patients, possibly tied to recurring severe infections (233). Jensen et al. discovered that a reduction in NK cells correlates with a worse sepsis prognosis (234), which is consistent with a previous study by Giamarellos-Bourboulis et al. showing that severe Gram-negative sepsis patients with increased NK cells survived longer that those patients with relatively lower NK cells (235).

Accumulating evidence indicates that high levels of lactate contribute to impaired function and decreased numbers of NK cells in both inflammatory diseases (236) and cancers (237–241). Using a murine cytomegalovirus (MCMV) model of infection, Dodard et al. found that lactate, independent of acidification, preferentially induces cellular apoptosis of tissue resident NK (trNK) cells when compared with conventional NK (cNK) cells in the liver (236). In addition, mechanistic studies revealed that mitochondrial fitness is impaired in trNK cells in comparison to cNK cells, which intensifies the cytotoxicity of lactate to trNK cells (236). In agreement, lactate-induced apoptosis of NK cells via enhancing mitochondrial stress is observed in colorectal liver metastasis (CRLM) tumors (237). Moreover, lactate is reported to decrease nuclear factor of activated T cells (NFAT), impairing NFAT-dependent IL-2, which is necessary for NK cell function (238, 242, 243). This deters NK cell activation and reduces IFN-γ, hindering immune surveillance in cancers (238). Downregulation of MCT4, a lactate transporter, restore the function of NK cells and the expression of cytokines (239). Lactate damages NK cell cytolytic function (240) via the SIX1/LDHA axis (241). Notably, this study found that in the lactate-rich malignant pleural fluid, forkhead box P3 (Foxp3)+ NKT-like cells showed increased histone lactylation at the Foxp3 promoter site, reduced by the lactate transporter inhibitor 7ACC2 (244). The above studies of lactate-NK cell associations have primarily concentrated on cancers, it may not be directly applicable to sepsis. However, given the versatile nature of protein and lactate localization within cells, these mechanisms should not be disregarded.

### 4.4 Dendritic cells

As a pivotal antigen-presenting cell (245), dendritic cells (DC) rely on elevated glycolysis for activation, leading to substantial lactic acid production (246). They express high levels of GPR81, enabling lactate to bind and subsequently suppress MHC-II expression (247) and impacting their maturation and differentiation (248–250). Consistent with this, dendritic cells increasingly show immunological tolerance towards pathogens in sepsis with elevated levels of lactate (251). Lactate downregulates C-C chemokine type 7 (CCR-7) (252), a migration molecule, and CD11c, a DC marker (253). Furthermore, lactate can hinder DC functionality by promoting Ca2^+^ mobilization to regulate IFN-α expression and reducing the levels of cAMP, IL-6, IL-12, and type I IFN in a GPR81-dependent mechanism (254). It is reported that DCs are a major source of IL-10 in infectious disease (255). IL-10 expression defines an immunosuppressive DC population (256–259). DCs exposed to high lactate environment express more IL-10 (260–263), suggesting that lactate induces an immunosuppressive phenotype of DCs. Interestingly, genetic depletion of lactate receptor GPR81 not only suppresses IL-10 production in DCs but also boosts the production of pro-inflammatory cytokines (IL-6, IL-1β and IL-12) in DCs, which protects against experimental colitis (264).

Beyond GPR81-dependent mechanisms, lactate influences DC function by altering antigen presentation and cross-priming of CD8+ T cells (265). Consistent with this, dysfunctional dendritic cells reduce T cell activation, emphasizing its role in sepsis-associated immune paralysis (251). In addition, lactate inhibits the polarization of monocytes into DCs (250, 266). Moreover, it has been shown that lactate reprograms the metabolism of DCs, resulting in reduced glycolysis and increased fatty acid oxidation (FAO) (193, 267). These may be important reasons for the decrease in the number of DCs in sepsis with elevated lactate levels. DC function in sepsis patients is closely linked to their specific microenvironment (268). It has been shown that preventing apoptosis in dendritic cells during sepsis have the potential to improve survival (269). With the advancement of DC-related sepsis clinical trials, exploring lactate could be worthwhile. These multifaceted roles of lactate underscore its significant impact on DCs in sepsis.

### 4.5 Neutrophils

Neutrophils are one of most important components of cellular innate immunity (270) and they make up the majority of bone marrow-derived white blood cells (271). They are the first cells to reach sites of infection and provide initial support before adaptive immune responses activate (272). Neutrophil dysregulation in sepsis at the early stage involves not only an increase in immature/nonfunctional neutrophils in the blood but also exacerbates sepsis pathology through free radical oxygen production (273). These functions are dependent on glycolysis (78, 274). The most notable characteristic of neutrophils is their phagocytic function, for which lactate can supply the required ATP. It is possible lactate may also enhance this function via PI3K/Akt signaling (275). Also, treatment with both endogenous and exogenous lactate enhances the ability of neutrophils to form neutrophil extracellular traps (NETs) (276). Wen et al. reviewed the possible mechanisms of NET contribution to sepsis and noted that similar extracellular traps may exist for macrophages, dendritic cells, mast cells, eosinophils, and basophils (277). Also, lactate buildup in sepsis can reduce neutrophil apoptosis by modulating the MCT1/PD-L1 pathway (278).

In terms of cell migration, lactate promotes expression of CXCL1 and CXCL2, which are neutrophil mobilizers, and increases bone marrow vascular permeability by GRP81 signaling to help neutrophil migration (80). Chowdhury et al. revealed different mechanisms of how lactate promotes neutrophil migration and worsen vascular injury by AKT/HIF-1α/LDHA signaling (270).

Some clinical research indicates that L-lactate level in sputum was positively correlated with neutrophil count (279–281), making lactate a potential biomarker of lung inflammation. Furthermore, the relationship between neutrophil count and concentration of plasma lactate, neutrophil-to-lymphocyte ratio, and the concentration of plasma lactate can predict the outcomes in patients with sepsis (282–284).

## 5 The promise of targeting lactate metabolism and lactate-associated signaling

Currently, the lack of effective treatments leaves a critical gap in the management of sepsis/septic shock. As discussed above, it has become clear that lactate is not merely a byproduct of anerobic metabolism but is also extensively involved in modulating immune responses in sepsis. This insight paves the way for new therapeutic avenues by targeting lactate metabolism and lactate-associated signaling pathway in sepsis.

Direct inhibition of lactate production has been evaluated in clinical trials, especially in the field of cancer. This approach specifically targets metabolic pathways involved in lactate synthesis, with a primary focus on LDH, a key enzyme in this process. Gossypol (AT-101), a potent LDHA inhibitor, has been evaluated in treating various types of cancers in Phase I, Phase II and single arm and randomized trials (285, 286). Within these trials, a modest benefit was observed both in monotherapy and in combination with chemoradiation therapies (285, 287–290). In addition, CHK-336, a first-in-class orally active LDHA inhibitor, has been assessed for its tolerability, safety, and pharmacokinetic (PK) profile in healthy volunteers (Phase I, NCT05367661, Chinook Therapeutics Inc.). However, no clinical trials have been conducted to evaluate the safety, tolerance, and efficacy of LDHA inhibitors in sepsis/septic shock. Given that lactate is ubiquitously produced by nearly all cells, suppressing LDHA could lead to unpredictable and potentially adverse effects in sepsis. In contrast to the direct suppression of lactate production, enhancing lactate clearance, via hemofiltration and renal replacement therapy, offers a more balanced approach to manage elevated lactate levels, particularly in conditions like sepsis where systemic metabolic demands are heightened (291–294).

This review highlights several classic signaling pathways targeted by lactate that have long been the focus of basic and clinical research, leading to the development of potential therapies such as HMGB1 inhibitors, MIF inhibitors, MAPK inhibitors, NF-κB inhibitors, Sirtuin-1 activators, and antioxidants. Among these targets are also membrane receptors, such as the TLR4 antagonist TAK-242 (295), GPR81 agonist 3,5-DHBA and antagonist 3-OBA (21, 23, 54), and MCT antagonist (100). And drugs targeting NETs with therapeutic potential for sepsis, such as small polyanions, are also being evaluated in sepsis-related clinical trials (Phase II, NCT06548854, Grand Medical Pty Ltd). Additionally, the observation that lactate elevates the expression of PD-1 and CTLA-4 in Treg cells may provide insights into whether lactate should be measured in clinical trials of immune checkpoint inhibitors for sepsis. However, the potential benefits of targeting these lactate-associated pathways could be compromised by the systemic nature of sepsis/septic shock.

It is noteworthy that cellular therapies using mesenchymal stem cells (MSCs) have been initiated in clinical trials for sepsis (296, 297). It is worth considering whether cell transfer of genetic modified immune cells (targeting MCTs, GPR81 or lactate-associated signaling) could overcome lactate-induced immunosuppression (298–300). We and others have reported that MCT-mediated lactate uptake and lactate/GPR81-dependent signaling are potentially involved in regulating immune cell responses in murine polymicrobial sepsis (21, 23, 80). Additionally, preclinical studies have demonstrated that the lactate/GPR81 axis plays a significant role in promoting cancer cachexia. This finding is further supported by clinical observations in lung cancer patients, where elevated serum lactate levels correlate with disease progression (301). Persistent inflammation, immunosuppression, and catabolism syndrome (PICS) can develop in patients who survive initial sepsis (302). Further research is needed to determine if lactate drives PICS similarly to cancer cachexia and if this can be confirmed in the context of sepsis/septic shock.

## 6 Conclusion

Lactate, initially deemed a metabolic waste, has emerged as a key player in regulating immune cells and inflammation. The role of lactate has been particularly scrutinized in the context of sepsis, where it serves as a crucial biomarker for monitoring patient status. Despite extensive research, the specific effects of lactic acid on different immune cells, such as B lymphocytes, remain unclear, highlighting a gap in our understanding of its broader impacts on the immune system. However, lactate-guided resuscitation leaves much to be desired. Understanding lactate as part of a complex metabolic network, closely linked to various immune cell responses in sepsis, shifts the focus away from viewing it solely as a therapeutic target. The mechanisms through which lactic acid influences receptors like GPR81 and GPR132 are also not thoroughly understood, highlighting a need for further investigation to pinpoint its precise roles and regulatory mechanisms in immune function. In sepsis, the relationship of lactate with immunosuppression is of significant interest, with definitive mechanisms yet to be established. Better understanding the influence of this molecule on immune cell function in sepsis could greatly enhance the effectiveness of immune-related clinical trials for this condition.
